# Beyond Aesthetics: A Matched‐Controlled Study of Static and Dynamic Foot Characteristics of 83 Professional Ballet Dancers

**DOI:** 10.1002/jfa2.70207

**Published:** 2026-09-15

**Authors:** Tabea Arens, Mareike Sproll, Anja Hauschild, Christoph Kniewasser, Luisa Helfrich, Luisa Kosch, Astrid Zech

**Affiliations:** ^1^ Department of Human Movement and Training Science Institute of Human Movement Science University of Hamburg Hamburg Germany; ^2^ Center for Rehabilitation and Sports Medicine BG Klinikum Hamburg Hamburg Germany; ^3^ Department of Sport Science Friedrich Schiller University Jena Jena Germany

**Keywords:** arch index, ballet, dancers, hallux valgus, pedobarography

## Abstract

**Background:**

Professional ballet dancers are exposed to substantial repetitive loading of the lower extremities particularly the feet. The aesthetic and technical demands of ballet may lead to morphological adaptations overtime. This study aimed to compare static and dynamic foot characteristics between professional dancers and active controls.

**Methods:**

Eighty‐three professional ballet dancers (44 female, 39 male) and sex‐matched controls were assessed for static foot size (length and width) and arch parameters in sitting and standing using a caliper. Dynamic arch index, foot size, hallux angle, and foot progression angle were recorded with a pedobarography platform. Linear mixed models were computed separately by sex and condition (sitting, standing, and walking) with group (dancer vs. control) as fixed effect and controlled for age and BMI. The proportions of dancers versus controls with a hallux valgus angle exceeding 15° were analyzed with chi‐square tests for both sexes, and the influence of age, group (dancer vs. control), and sex were analyzed using a binary logistic regression model.

**Results:**

The dancers' static arch height index was higher, and their forefeet were wider than in the controls in both sitting and standing conditions (*p* < 0.001), whereas only female dancers showed a greater dynamic foot progression angle (*p* = 0.003) than the controls. Logistic regression revealed a nonsignificant, 2.8‐fold higher likelihood of a hallux angle of 15° or greater in dancers.

**Conclusion:**

Ballet dancers demonstrate different static and dynamic foot characteristics compared to controls. The results underline the need for further longitudinal studies investigating how sex‐specific loading patterns as well as ballet footwear affect the development of foot structure and function over time.

## 1 Introduction

**FIGURE 1:**
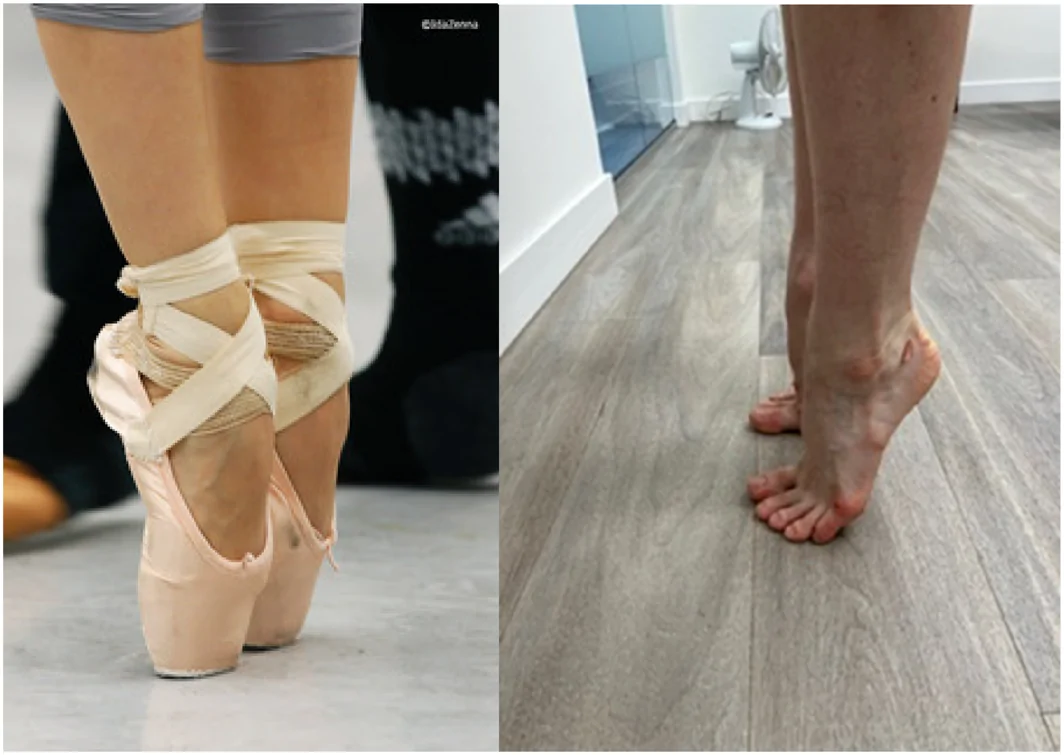
Dancer's feet on pointe (left) Ida Zenna and demi‐pointe (right) Anja Hauschild.

Professional dancers, particularly ballet dancers, are exposed to substantial biomechanical demands arising from both technical and aesthetic requirements [1]. Daily work schedules may encompass up to 8 hours of physical activity and a mean of five jumps every minute per performance [2]. Specific techniques such as turnout, demi‐pointe, and pointe work (Figure 1) [3], further expose the lower extremities—particularly the feet—to repetitive loading and high mechanical stress [1, 4]. However, it remains unclear whether long‐term exposure leads to morphological adaptations of the foot's architectural characteristics compared to nondancers.

Historically, the aesthetic requirements of ballet have shaped not only training but also dancer selection, favoring specific foot morphologies. An “ideal” ballet foot is characterized by high flexibility in dorsiflexion and plantarflexion as well as a pronounced longitudinal arch, to execute correct technique especially when pointing the foot [3]. However, an excessively high arch (pes cavus) might impair shock absorption during landing [4] due to its biomechanical restriction of ankle dorsiflexion. Previous studies reported higher arch heights in dancers compared to nondancers typically based on static footprint analyzes [3]. Moreover, the foot progression angle, defined as the angle between the foot's longitudinal axis and the walking direction [5], reflects the net rotational orientation of the entire lower extremity during gait. As turnout in ballet requires coordinated external rotation across multiple lower limb joints, its habitual practice may carry over into increased out‐toeing during natural gait [6]. Although dancers show increased hip extension and abduction during walking compared to nondancers [7], no study has yet quantified foot progression angles during natural gait in ballet dancers, and it remains unknown whether dance‐specific turnout pattern, once habitually trained, generalize to movement tasks outside the studio, such as natural walking.

During walking, the hallux contributes substantially to propulsion, bearing up to 29% of body weight under the first metatarsal head [8]. Hallux valgus—defined as a lateral deviation of the first toe combined with a medial deviation of the first metatarsal and clinically diagnosed if the angle exceeds 15° [9]—is frequently reported in both the general population and among dancers [10]. Alterations in hallux alignment have been linked to reduced push‐off forces and a redistribution of plantar loads toward the lesser metatarsals [11, 12, 13], potentially compromising the efficient propulsion and load distribution required for dance‐specific movements such as jumping and pointe work. General risk factors include female sex and high‐heeled footwear [14], whereas dance‐specific contributors may include restrictive footwear such as ballet slippers and pointe shoes, deficient demi‐pointe and pointe technique, and compensated turnout patterns [15]. Given these dance‐specific exposures, professional ballet dancers may represent a population at particular risk for hallux valgus and its functional consequences.

Despite the clinical relevance of hallux valgus and the functional importance of foot morphology and progression angle for dance performance, the systematic assessment of these characteristics in professional ballet dancers remains substantially understudied. The study aims to analyze and compare parameters of general and clinically relevant foot characteristics between professional ballet dancers of three major German ballet companies and healthy controls. Given that the female sex constitutes an established risk factor for hallux valgus and that dancers are exposed to additional, sex‐specific dance characteristics such as dancing on pointe shoes, all analyses were performed separately to account for potential sex‐specific differences in foot adaptation. To account for the flexible adjustment of foot structures to movement conditions, tests were performed under static (standing and sitting) and dynamic walking conditions. We hypothesized that (1) dancers would show a higher longitudinal arch in both static and dynamic conditions (2), dancers, especially females, would exhibit a greater hallux valgus angle, and (3) dancers would display a greater foot progression angle during gait.

## 2 Methods

### 2.1 Participants

Dancers from three professional dance companies of German opera houses and state theaters participated in the in‐house health & performance screenings between November 2024 and August 2025 into which the measurements for this study were embedded. Participation was voluntary, and tests took place during working hours. Dancers were eligible for inclusion if they were free of any lower extremity injury requiring medical attention and able to participate in regular dance class at the time of the screening. Data from dancers were compared with data from healthy, sex‐matched, physically active individuals (sport students) recruited among the university's sports students and staff population. All participants received written study information and provided informed consent prior to participation. Ethical approval for this study was granted by the Ethics Commission of the Friedrich Schiller University Jena (FSV 24/108 and FSV 23/076).

### 2.2 Static and Dynamic Foot Measurements

**FIGURE 2:**
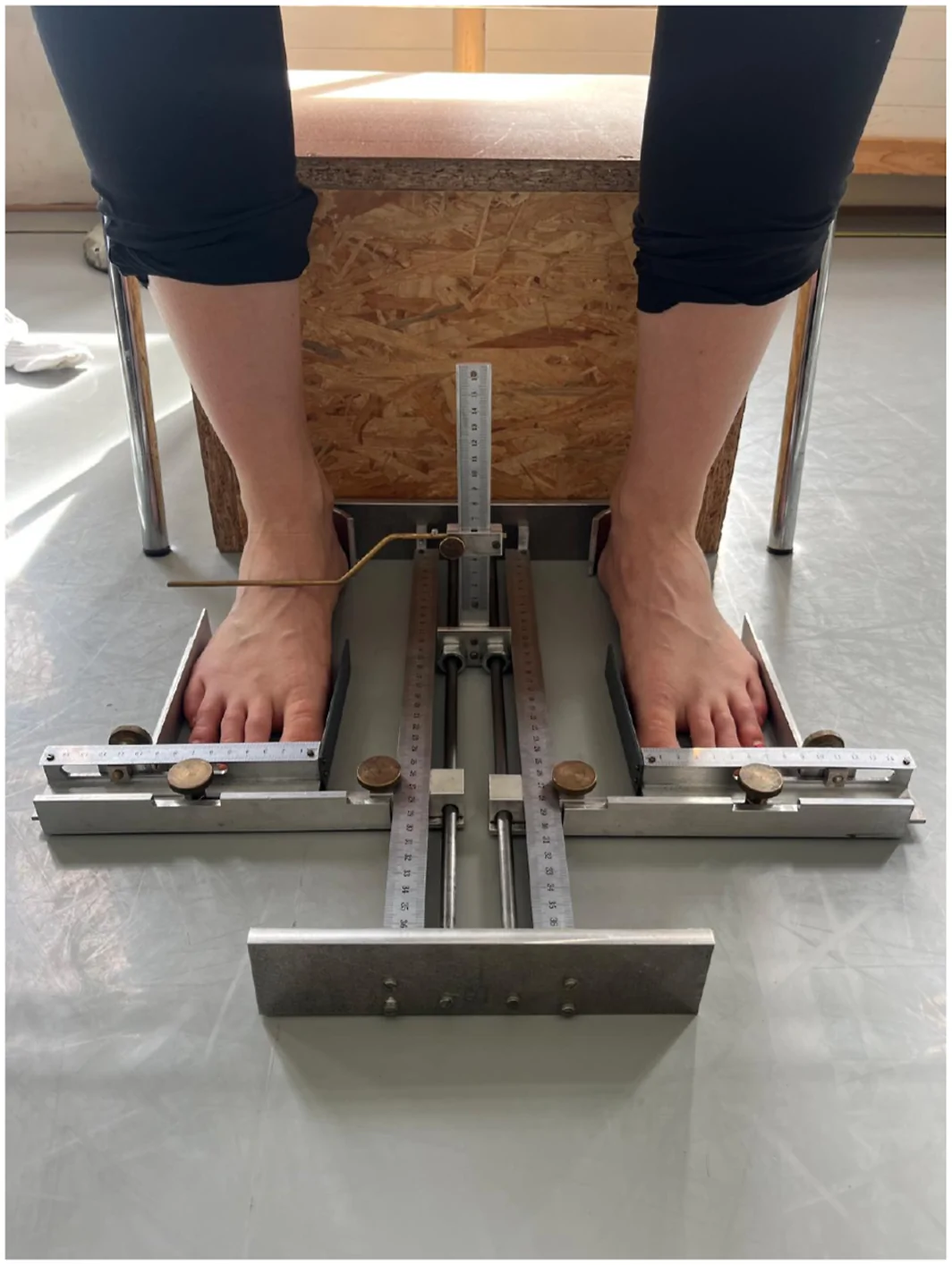
Caliper used for static foot measurements during sitting and standing.

Static foot measurements were measured using a specifically constructed caliper (Figure 2), which was found to have excellent reliability (0.96–0.99 intrarater reliability, 0.98–0.99 interrater reliability) for measuring heel‐to‐toe length and excellent reliability (0.94 intrarater reliability, 0.99 interrater reliability) for the arch height index [16].

Data collection was performed in two positions: sitting, equaling approximately 10% of the body weight on each foot, and standing [17]. In both positions, heel‐to‐toe length, forefoot width at the widest point of the forefoot, dorsum height at 50% of the heel‐to‐toe length, and the arch height index, calculated as dorsum height/heel‐to‐toe length, were measured. All measurements are displayed in cm.

Dynamic foot measurements were obtained using a high‐resolution capacitive pressure pedobarographic device (*Emed‐n50 platform, Novel GmbH, Munich, Germany*) with a total of 6080 sensors (4 sensors/cm^2^, area: 475 × 320 mm, sampling rate: 50 Hz). The Emed system was found to have an accuracy of ± 5%, a test‐retest reliability interclass coefficient (ICC) of > 0.8 [18]. A reliable two‐step protocol was employed [19] where participants took one step on a wooden platform before reaching the integrated device at the middle of the wooden walkway (600 × 3600 mm). Prior to collection, each participant's individual starting position was determined by having them walking two steps forward from the middle of the platform towards the end of the wooden walkway. This point served as the starting point for all subsequent trials. From this position, participants walked at a self‐selected, comfortable pace across the platform without consciously targeting it [17], continuing to the end of the walkway before returning to the starting position via a lateral path. Familiarization trials were performed for each leg. Three valid trials per leg were recorded, and trials were repeated in case of misplacement on the platform borders. For each outcome variable, the mean of the three valid trials was used. Following outcome variables were collected: dynamic heel‐to‐toe length, dynamic forefoot width, dynamic foot progression angle, according to the manufacturer defined as the angle between the axis and a vertical line parallel to the platform *Y* axis [20] in degrees, dynamic arch index, calculated as the ratio of the midfoot contact area to the total foot contact area excluding the toes [17, 21] and dynamic hallux angle, determined “by intersection of the bisection of metatarsal 1—metatarsal 2 angle and axis, which is drawn through intersection of this bisection with line separating toes from metatarsals and center of big toe” [20].

The participants were not informed about the specific hypotheses or targeted outcome variables of the foot assessments to minimize expectancy or performance bias during testing. Data were collected by specifically trained personnel, and all measurements were taken barefoot.

### 2.3 Statistical Analysis

The static measurements were manually entered in Excel (Version 16.97.2, Microsoft 365), and all outcome variables were subsequently analyzed in RStudio (Version 2024.12.1+563, Posit Software, PBC) [22]. Sex distribution was equalized by stratified random sampling within the control group, resulting in equal sample sizes. The entire analysis was performed for the male and female sexes separately to acknowledge sex‐specific differences. Descriptive statistics (mean ± standard deviation) are displayed for dancers and controls. Means and standard deviations for all variables are presented in plotted graphs. The alpha level for significance was set at *p* < 0.05. Missing data were handled with listwise exclusion per analysis.

To evaluate group differences in foot parameters, linear mixed models (LMMs) were fitted using the lme4 package [23] separately for male and female participants for three conditions (sitting, standing, and dynamic), resulting in six model blocks in total (2 sexes * 3 conditions). Within each block, one model was fitted per outcome variable (heel‐to‐toe length, forefoot width, arch height index for both static sitting and standing; and heel‐to‐toe length, forefoot width, arch index, foot progression angle, and hallux angle for walking). Each model included group (dancer vs. control) as a fixed effect, the participants' ID as a random intercept to account for repeated measurements from the left and right foot, age, and the body mass index (BMI) as covariates. Missing data were handled by listwise exclusion per model. Model assumptions were inspected using the performance package [24], influential observations were identified via Cook's distance and leverage plots, and cases flagged as potential outliers were inspected individually and retained in the final analysis. Model outputs included fixed effect estimates, 95% confidence intervals (CIs), t‐ and *p*‐values. Standardized effect sizes were calculated as Hedges' g derived from estimated marginal means contrasts using the emmeans package [25]. The control group served as the reference level. Bonferroni corrections for multiple testing were applied within each condition (sitting, standing, and dynamic) and sex.

To assess the prevalence of hallux valgus deformity, the proportion of participants with angles ≥ 15° was displayed for each group and sex, and differences between the groups were analyzed with chi‐square (χ^2^) tests for both sexes. A binary logistic regression model was fitted to examine the influence of age, group (dancer vs. control), and sex on the likelihood of exhibiting an increased hallux angle. Odds ratios (OR), 95% CI, and *p*‐values were reported.

## 3 Results

Data were collected from 83 dancers (44 female and 39 male) and 83 sex‐matched controls. Following the loss of three recordings, 163 files per group (dancers and controls, comprising both right and left feet) entered the analysis. Demographic characteristics of the dancers and the control groups are summarized in Table 1. Dancers of both sexes were significantly older than their control counterparts. Additionally, they had a lower body weight, and male controls were taller than the group of dancers. Participation rate in the individual companies was 52.4%, 86.8%, and 89.5%.

**TABLE 1:** Participants' characteristics.

	Male		*p* value	Female		*p* value
	Dancer, mean (± SD) *N* = 39	Control, mean (± SD) *N* = 39		Dancer, mean (± SD) *N* = 44	Control, mean (± SD) *N* = 44	
Age, years^a^	27.6 (6.2)	22.4 (2.3)	< 0.001	26.1 (5.6)	22.7 (3.4)	0.005
Body height, cm^a^	176.7 (5.0)	181.6 (9.8)	0.007	166.0 (4.5)	167.9 (6.6)	0.064
Body weight, kg^a^	68.1 (6.4)	81.0 (11.5)	< 0.001	52.0 (4.9)	64.0 (7.1)	< 0.001

^a^ Wilcoxon rank sum test. All values are expressed as mean ± standard deviation.

**FIGURE 3:**
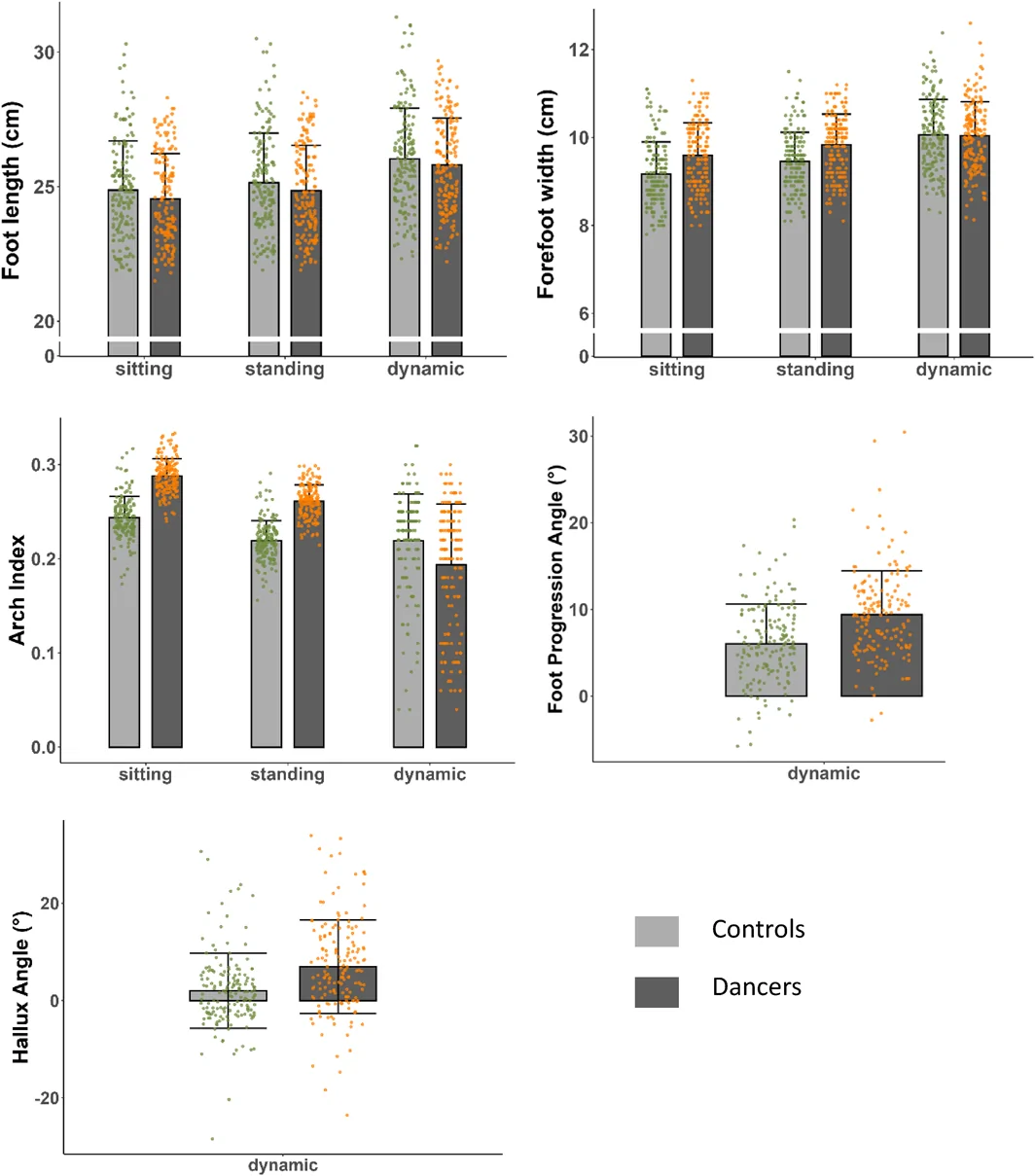
Group differences between Controls (left) and Dancers (right) for foot length (cm), forefoot width (cm), and arch index across the three different positions sitting, standing, and dynamic. Foot progression angle (°) and hallux angle (°) are only presented in the position measured, dynamically.

The mean values (± standard deviation) for static and dynamic measurements are summarized in Figure 3, accompanied by the mixed model results in Tables 2 and 3. In the static sitting and standing positions, both male and female dancers differed in arch height index and forefoot width compared to controls after adjusting for BMI and age. On average, male dancers had a 0.05 units higher arch height index than male controls in sitting and standing with a very large effect size (*p* < 0.001, Hedges' g 6.429 and 5.709) and a 0.6 cm wider forefoot compared to male controls (*p* < 0.001, Hedges' g 2.437 and 2.570). In males, no significant group differences were observed for heel‐to‐toe length in the static positions and for all variables in the dynamic positions. Even though nonsignificant after Bonferroni correction, the difference in foot progression angle between male dancers and controls showed a very large effect size with a Hedges' g of 1.125 (10.44° in male dancers vs. 8.16° in male controls, *p* = 0.099). Large to very large effect sizes were also observed comparing foot length with a Hedges' g of −0.935 (Estimate 0.306), and arch index with a Hedges' g of −0.557 (Estimate 0.014), even though both were nonsignificant. In females, dancers had an on average 0.54–0.57 cm larger forefoot width compared to controls (Hedges' g 2.059, and 2.155, respectively, *p* ≤ 0.001), and a higher arch height index by 0.05, respectively, 0.04 units (Hedges' g 3.703 and 3.467 respectively, *p* < 0.001). In the dynamic condition, female dancers showed significantly greater foot progression angle (Estimate 3.399, *p* = 0.01) compared to female controls. Further, female dancers showed a hallux angle on average of 5.53° greater compared to female controls with a very large effect size (Hedges' g 1.039) but without statistical significance after Bonferroni correction (*p* = 0.129).

**TABLE 2:** Results of the linear mixed models comparison between male dancers and controls adjusted for BMI and age.

Variable	Mean dancer ± SD	Mean control ± SD	Estimate	95% CI	*t*	*p*	*p*, Bonf.	Hedges' g	95% CI
Static (sitting)									
Heel‐to‐toe length, cm	26.07 (1.05)	26.22 (1.54)	0.3119	[−0.47; 1.09]	0.7829	0.4359	1.0000	−1.545	[−5.475; 2.386]
Forefoot width, cm	10.15 (0.49)	9.71 (0.61)	−0.5885	[−0.89; −0.28]	−3.7991	0.0003	0.0009	2.437	[1.127; 3.746]
Arch height index	0.28 (0.01)	0.24 (0.02)	−0.0500	[−0.06; −0.04]	−11.4587	0.0000	0.0000	6.429	[5.075; 7.783]
Static (standing)									
Heel‐to‐toe length, cm	26.34 (1.06)	26.54 (1.58)	0.2901	[−0.50; 1.08]	0.7162	0.4759	1.0000	−1.326	[−5.013; 2.361]
Forefoot width, cm	10.31 (0.42)	9.93 (0.54)	−0.5591	[−0.82; −0.30]	−4.1812	0.0001	0.0003	2.570	[1.309; 3.831]
Arch height index	0.26 (0.02)	0.22 (0.02)	−0.0517	[−0.06; −0.04]	−11.1902	0.0000	0.0000	5.709	[4.487; 6.931]
Dynamic (walking)									
Foot length, cm	27.28 (1.13)	27.39 (1.65)	0.3059	[−0.52; 1.13]	0.7248	0.4706	1.0000	−0.935	[−3.506; 1.635]
Forefoot width, cm	10.60 (0.57)	10.63 (0.61)	−0.0927	[−0.42; 0.23]	−0.5595	0.5773	1.0000	0.265	[−0.678; 1.207]
Hallux angle, °	3.14 (8.95)	0.91 (7.08)	−1.2525	[−5.80; 3.30]	−0.5393	0.5912	1.0000	0.259	[−0.697; 1.214]
Foot progression angle, °	10.44 (5.93)	8.16 (4.55)	−3.5613	[−6.50; −0.62]	−2.3760	0.0198	0.0990	1.125	[0.172; 2.077]
Arch index	0.21 (0.06)	0.23 (0.05)	0.0144	[−0.02; 0.04]	0.9533	0.3433	1.0000	−0.557	[−1.722; 0.608]

**TABLE 3:** Results of the linear mixed models comparison between female dancers and controls adjusted for BMI and age.

Variable	Mean dancer ± SD	Mean control ± SD	Estimate	95% CI	*t*	*p*	*p*, Bonf.	Hedges' g	95% CI
Static (sitting)									
Heel‐to‐toe length, cm	23.24 (0.84)	23.71 (1.12)	0.6976	[0.10; 1.30]	2.2850	0.0248	0.0744	−3.094	[−5.806; −0.381]
Forefoot width, cm	9.10 (0.53)	8.71 (0.46)	−0.5422	[−0.82; −0.26]	−3.7952	0.0003	0.0009	2.059	[0.958; 3.160]
Arch height index	0.29 (0.02)	0.25 (0.03)	−0.0473	[−0.06; −0.03]	−6.8211	0.0000	0.0000	3.703	[2.552; 4.853]
Static (standing)									
Heel‐to‐toe length, cm	23.59 (0.89)	23.95 (1.05)	0.5627	[−0.03; 1.15]	1.8666	0.0654	0.1962	−2.684	[−5.558; 0.190]
Forefoot width, cm	9.41 (0.60)	9.05 (0.46)	−0.5683	[−0.87; −0.27]	−3.6797	0.0004	0.0012	2.155	[0.968; 3.343]
Arch height index	0.26 (0.02)	0.22 (0.02)	−0.0416	[−0.05; −0.03]	−6.7794	0.0000	0.0000	3.467	[2.383; 4.550]
Dynamic (walking)									
Foot length, cm	24.52 (0.95)	24.87 (1.16)	0.4856	[−0.15; 1.12]	1.4940	0.1389	0.6945	−1.447	[−3.379; 0.485]
Forefoot width, cm	9.56 (0.59)	9.58 (0.61)	−0.1649	[−0.49; 0.16]	−0.9841	0.3279	1.0000	0.442	[−0.453; 1.338]
Hallux angle, °	9.93 (9.23)	2.94 (8.12)	−5.5287	[−10.30; −0.76]	−2.2707	0.0257	0.1285	1.039	[0.122; 1.956]
Foot progression angle, °	8.43 (4.14)	4.23 (3.81)	−3.3993	[−5.48; −1.32]	−3.1973	0.0019	0.0095	1.189	[0.438; 1.939]
Arch index	0.18 (0.07)	0.21 (0.05)	0.0090	[−0.03; 0.04]	0.5142	0.6084	1.0000	−0.321	[−1.564; 0.921]

**FIGURE 4:**
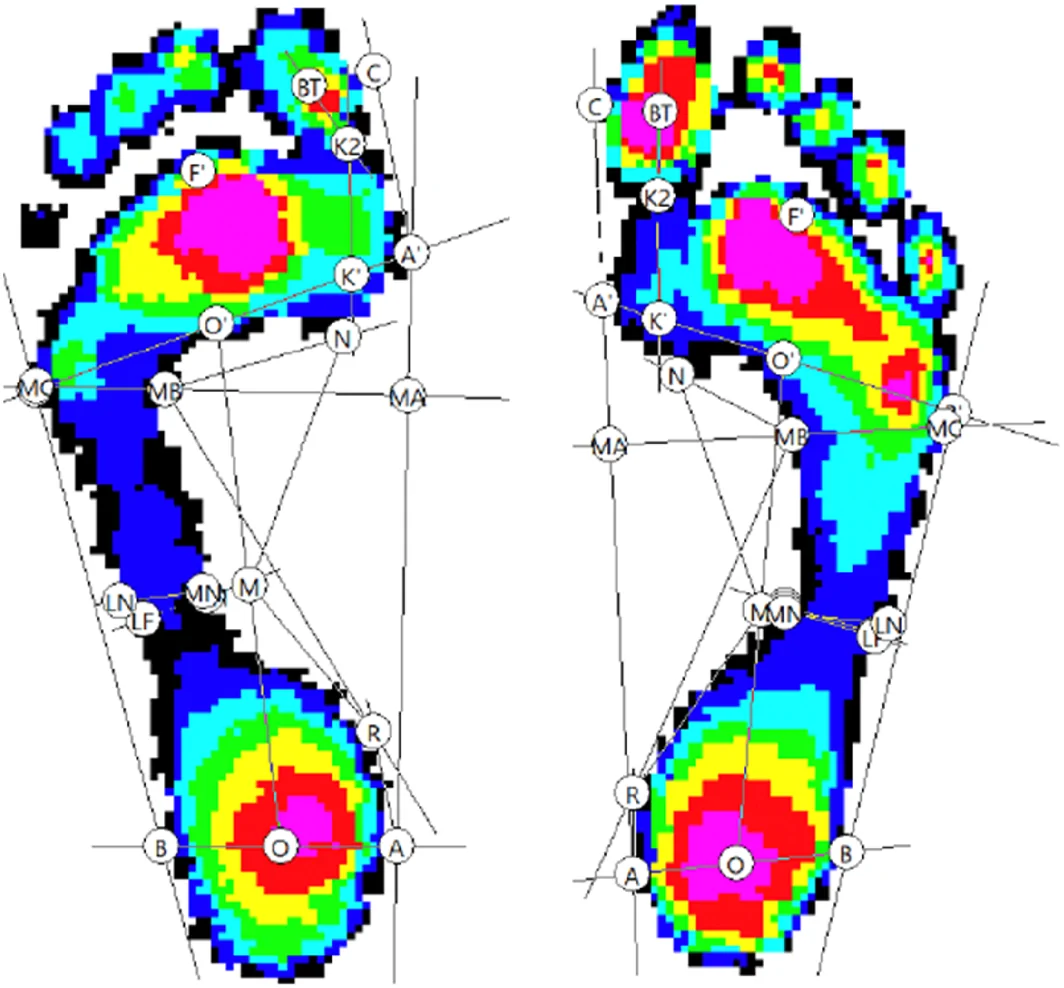
Emed footprints of two different participants with hallux angles of 33.3° (left) and 2.04° (right).

A footprint showing a hallux angle of 33.3° versus one with 2.04° can be seen in Figure 4. A hallux angle ≥ 15° was present in 30 females and 11 males. The prevalence was significantly higher in female dancers (*n* = 23) compared to female controls (*n* = 7, *p* = 0.003) but did not differ between male groups (Table 4). Separate binary logistic regression models were fitted for male and female participants to examine the influence of age, BMI, and group (dancer vs. control) on the likelihood of exhibiting a hallux angle ≥ 15° (Table 5). In neither sex was dancer status a significant predictor after adjustment for age and BMI: female dancers showed an approximately three‐fold higher likelihood of hallux valgus than female controls, but this difference did not reach statistical significance (OR = 2.96, 95% CI [0.842; 12.221], *p* = 0.108), no corresponding association was found in males (OR = 1.50, 95% CI [0.196; 13.037], *p* = 0.698).

**TABLE 4:** Chi‐square (*X* ^2^) comparison of hallux angles ≥ 15° by sex and group.

Sex	Group	Total feet	*n* ≥ 15° (%)	*n* < 15° (%)	*p* value (*X* ^2^)
Female	Control	88	7 (8%)	81 (92%)	
Female	Dancer	88	23 (26.1%)	65 (73.9%)	0.003
Male	Control	75	4 (5.3%)	71 (94.7%)	
Male	Dancer	75	7 (9.3%)	68 (90.7%)	0.533

**TABLE 5:** The results of the logistic regression for hallux valgus (≥ 15°), adjusted for age and BMI, by sex.

Predictor	OR	95% CI	*p*
Male			
Age	1.090	[0.951; 1.252]	0.2142
BMI	1.177	[0.830; 1.642]	0.3390
Group	1.501	[0.196; 13.037]	0.6983
Female			
Age	1.020	[0.939; 1.103]	0.6310
BMI	0.934	[0.721; 1.191]	0.5911
Group	2.961	[0.842; 12.221]	0.1082

## 4 Discussion

### 4.1 Summary of Key Results

This study identified considerable differences in both static and dynamic foot characteristics between professional ballet dancers and matched controls. In static sitting and standing conditions, male and female dancers demonstrated higher foot arches and wider forefeet than controls. During walking, there was one sex‐specific group difference identified. Female dancers exhibited a larger foot progression angle and showed a nominally greater hallux angle compared to controls, although the latter difference did not remain significant after correction for multiple comparisons. Female dancers also showed a higher unadjusted prevalence of a hallux angle of 15° or greater than female controls and all male participants; however, this association was attenuated after adjusting for age and BMI.

### 4.2 Forefoot Width and Heel‐to‐Toe Length

In general, the heel‐to‐toe length and forefoot width measured in dancers and controls fall in line with data from a previous study [3]. Although heel‐to‐toe length did not differ between dancers and controls of either sex, forefoot width was significantly wider in this cohort of dancers under static conditions by 0.5–0.6 cm, even though, besides excellent measurement reliability [18], the observed group differences lie near or below measurement error. Previous authors reported a frequent noticing of the forefoot widening in classically trained ballet dancers [3], which might be due to multiple potential factors. First, the natural loading of the foot is influenced by pointe and demi‐pointe work. Especially in the latter, body weight is excessively loaded onto the forefoot and toes, concentrating load on the metatarsal head and first ray [3, 26, 27]. This static redistribution is compounded by the impact loading of jumping: ballet‐specific jumps generate peak vertical ground reaction forces reported between 1.4 and 9.6 times body weight [28]. Even though the net ground reaction forces decrease when wearing pointe shoes, as jump height decreases [29], the forces are probably transmitted through a markedly reduced forefoot contact area, further elevating local mechanical stress at the metatarsal region. Given that professional dancers perform up to 200 jumps per class [30], which, at professional level, takes place at five to 6 days per week, the forefoot is exposed to an exceptionally high cumulative dose of localized, repetitive loading. Further, this effect may be attenuated considering that most dancers start dancing at a very young age and even at preprofessional level face an accumulated activity of 30 hours per week [31]. Especially during the vulnerable phase of ossification in the developing bone, it has been postulated that the cumulative stress on the forefoot might influence specific bony adaptations [3] and, in this case, wider forefeet.

Second, ballet dancers are expected to have extraordinary balance abilities in various positions [32]. It has been reported that a larger foot size in general, but also wider forefeet specifically, is associated with superior balance performance, as the area of support increases [33]. Considering the large number of hours spent dancing when starting from a young age, an adaptation of the forefeet to meet the demands can be expected, even though Gorwa et al. [3] postulated that the forefoot width is not influenced by weekly training volume and career duration. Future longitudinal studies might have to differentiate an incidental finding from a true load‐induced adaptation of the foot.

In the dynamic condition, the previously observed group difference in forefoot width equalized. Forefoot width is described to progressively increase with load, as the intersegmental motion distal to the rearfoot increases to distribute load when walking [34]. Because static and dynamic forefoot width were assessed with different measurement devices, however, the magnitude of forefoot spreading between conditions cannot be directly compared in this study.

### 4.3 Arch Height

In static conditions, a greater arch height index (calculated as dorsum height divided by heel‐to‐toe length) was observed in both male and female dancers compared to the controls, indicating a higher longitudinal arch. This is consistent with previous studies reporting higher Clarke angles and navicular heights in professional dancers [3], collegiate dancers [35], and adolescent dancers [36]. A greater arch height has previously been reported to be associated with a greater activity level but also habitual barefoot or minimal footwear use [16, 17, 37], a mechanism that may be less applicable to ballet dancers given the often use of ballet slippers or pointe shoes. As Gorwa et al. [3] found no relationship between career duration or training volume and arch height, this argues against a purely load‐dependent adaptation mechanism specific to footwear exposure and instead lends support to a preselection effect, whereby dancers with a naturally high arch are preferably selected into professional training due to the aesthetic demands of the pointed foot position [38].

Generally, the dynamic arch index of the dancers is comparable to previously reported data of a habitually shod population [39] and, in contrast to the static measurements, did not differ between dancers and controls. A possible, yet speculative, explanation is that the reaction of arch index to load seems to differ by foot type. Generally, the arch index is expected to increase with increasing load, and thus the arch decreases [40]. As described by Kramer & Lautzenheiser [40], in the cavus foot type, a very high arch which is often found in dancers [3, 36], the arch varies more under increasing load compared to a more planus foot type. The latter tends to stiffen during walking compared to standing, as a result of greater muscle activation, leading to a rebuild of the arch [40]. This greater load‐dependent variability of the cavus foot type might therefore have contributed to the convergence of dynamic arch index values observed between dancers and controls despite the static differences. Even though the mean of female dancers seemed to be slightly lower than that of female controls and both male groups, a true morphological difference seems unlikely considering the measurement error of 0.06 [18] and the estimate of 0.009 in this cohort of female dancers and controls.

### 4.4 Foot Progression Angle

Female dancers exhibited greater out‐toeing during gait than female controls. This may reflect a transfer of the externally rotated turn‐out position, which is trained and maintained throughout the dancers' everyday life into natural gait. It was previously reported that the foot progression angle in gait is associated with a greater tibial torsion [41]. Literature comparing the tibial torsion of dancers versus nondancers is limited, and tibial torsion values reported across adult and adolescent ballet dancers vary considerably [42], suggesting that tibial torsion alone may not adequately explain differences in the foot progression angle during walking within this population.

Another, currently underexplored explanation concerns the knee external rotation as a compensational strategy used by dancers to extend functional turnout beyond what is achievable through hip external rotation alone [43, 44]. However, a direct link between compensatory loading, possible subsequent structural adaptation, and greater foot progression angle during walking was not analyzed in this study and remain speculative.

Nevertheless, previous research has also demonstrated substantial natural variability of the foot progression angle in healthy adults [18], and the between‐group difference of 3.39° therefore falls within the device's expected measurement range.

### 4.5 Hallux Valgus

The hallux valgus deformity is a well‐known problem in dancers and is frequently associated with pain [45], which might especially be present in pointe shoes due to the bunion on the medial head pressing against the restrictive pointe shoe, impaired balance, and reduced quality of life [46]. Altered gait loading patterns, including decreased push‐off capacity of the first ray and reduced plantar flexor contribution during propulsion [11], together with decreased strength of the toe flexors and the abductor hallucis muscle [47], may further compromise both walking efficiency and high‐demand tasks such as jumping, one of the key performance indicators of dance.

Whether dancers are more likely to develop a hallux valgus deformity than nondancers is controversially discussed, as previous study findings are inconsistent [48, 49, 50]. In the present study, 26% of the female dancers had a dynamic hallux angle of 15° or greater compared to only 8% in the controls. The prevalence of a hallux valgus seems lower in this cohort compared to previous literature both in dancers and controls [10]. However, in the meta‐analysis by Nix et al. [10], participants were between 18 and 65 years old and therefore considerably older than both dancers and controls in our cohort.

Generally, female sex is one of the main risk factors for the deformity [10, 47]. Even though nonsignificant, an average female dancer of this cohort had a hallux angle of 5.5° greater compared to an average female control with a rather large effect size of > 1. Nonsignificantly, female dancers in this cohort were 3‐times more likely to have a hallux angle greater 15° than controls. This tendency was not visible in males.

Dance‐specific loading characteristics are discussed as influencing factors for a deformity of the first ray though clear evidence remains lacking [51]. Footwear is generally discussed as one of the main influencing factors of a hallux valgus deformity. First, ballet‐specific footwear, such as slippers and pointe shoes, often deliberately worn a size smaller for aesthetic reasons [50], can increase valgus stress at the first metatarsophalangeal joint and promote lateral deviation of the hallux. Although a causal relationship has yet to be established, pointe shoe use is widely discussed as a risk factor [50] particularly given the high pointe exposure in professional training (estimated at 24h per week) [3]. Second, faulty turnout technique may exacerbate valgus stress [52]. When hip external rotation is limited, dancers tend to increase their turnout through hindfoot eversion, midfoot abduction, and foot pronation [43]. Maintaining this compensatory strategy requires the toes to grab into the floor, creating friction between foot and floor, and the tibia rotating internally [53], which again results in greater valgus forces on the first MTP joint [50]. Generally, the role of dance‐specific movements in the development of hallux valgus deformities needs to be further researched in prospective studies [52].

### 4.6 Limitations and Future Directions

This study has several limitations that should be considered when interpreting the results. First, hallux valgus was not radiographically confirmed; instead, the angle was derived solely from dynamic pedobarographic measurements. The commonly used cut‐off of 15° originates from radiographic studies [9], and it is currently unclear whether this threshold is directly transferable to dynamic pressure‐based measurements. Although the control group provides an important internal reference, future work should include a validation against radiographic gold standards to establish the accuracy and diagnostic relevance of pedobarographic hallux valgus measurements. Second, the generalizability of the results is limited to classical ballet dancers. Ballet involves pointe work and technical specifications such as turnout which are not found in every dance style. Therefore, the findings may not be transferable to the general dance population. Third, walking speed was not standardized, which may have introduced variability in dynamic parameters. Fourth, a selection bias might have occurred as not all dancers of each company chose to participate. Participation rate in the individual companies was 52.4%, 86.8%, and 89.5%, which is still considered a rather large sample size for a cohort as specialized as professional dancers. Fifth, the control group was matched to the dancers by sex only; detailed information on physical activity level and training volume was not systematically collected, limiting the ability to fully attribute observed differences to dance‐specific adaptations. Additionally, the control group was significantly younger than the cohort of dancers. Even though the analyses accounted for the difference in age, performing the analyses with an age‐matched group might provide further insight. To address these limitations, future research should investigate how children's feet develop under pointe work and long‐term ballet training, particularly in female dancers, who were affected by the hallux valgus deformity more often in this cohort. Furthermore, combining pedobarographic assessments with in‐shoe pressure measurements and radiographic imaging could provide more comprehensive insights into both functional loading and structural changes over time.

## 5 Conclusion

The foot characteristics of professional ballet dancers differ compared to nondancers. Although both sexes of ballet dancers had higher arches and wider forefeet than the control population, only female dancers showed a significantly greater foot progression angle and a higher prevalence of hallux angles exceeding 15° compared to controls. These findings highlight the need for longitudinal research examining how early training exposure, pointe work, and footwear interact with foot structure over time, and whether preventive strategies can mitigate long‐term structural adaptations or pathology.

## 6 Perspective

Previous research has shown that factors such as habitual loading and footwear use from an early age strongly influence the development of a natural arch and hallux valgus angles [37, 54]. In this cohort of professional ballet dancers, both males and females had higher static arch height indices and wider forefeet compared to active controls. These differences in foot characteristics likely reflect ballet‐specific adaptations of foot structure and function driven by training load, technique, and footwear constraints. Moreover, they are likely reinforced by preselection criteria in dance schools that favor dancers with a visibly high arch, considered aesthetically desirable [3]. Female dancers were further found to be more likely to present a hallux valgus deformity of 15° or greater, a condition known to be associated with reduced push‐off, altered plantar pressure distribution, impaired balance, and increased pain [11, 12, 13]—factors that can compromise performance. Therefore, in future studies, it should be aimed to longitudinally monitor young dancers to capture load‐dependent structural adaptations associated with the initiation and progression of pointe‐shoe work.

## Author Contributions

**Tabea Arens:** conceptualization, data curation, formal analysis, writing – original draft. **Mareike Sproll:** data curation, formal analysis, writing – review and editing. **Anja Hauschild:** conceptualization, data curation, writing – review and editing. **Christoph Kniewasser:** data curation, writing – review and editing. **Luisa Helfrich:** data curation, writing – review and editing. **Luisa Kosch:** data curation, writing – review and editing. **Astrid Zech:** conceptualization, supervision, writing – review and editing.

## Funding

The authors have nothing to report.

## Ethics Statement

Ethical approval for this study was granted by the Ethics Commission of the Friedrich Schiller University Jena (FSV 24/108 and FSV 23/076), and all procedures involving human participants were in accordance with the Helsinki Declaration.

## Consent

All participants received written study information and provided informed consent prior to participation.

## Conflicts of Interest

The authors declare no conflicts of interest.

## Data Availability

The data that support the findings of this study are available from the corresponding author upon reasonable request.
